# Adipose Tissue and Adrenal Glands: Novel Pathophysiological Mechanisms and Clinical Applications

**DOI:** 10.1155/2014/614074

**Published:** 2014-06-11

**Authors:** Atil Y. Kargi, Gianluca Iacobellis

**Affiliations:** Division of Endocrinology, Diabetes and Metabolism, Department of Medicine, University of Miami Miller School of Medicine, Miami, FL 33136, USA

## Abstract

Hormones produced by the adrenal glands and adipose tissues have important roles in normal physiology and are altered in many disease states. Obesity is associated with changes in adrenal function, including increase in adrenal medullary catecholamine output, alterations of the hypothalamic-pituitary-adrenal (HPA) axis, elevations in circulating aldosterone together with changes in adipose tissue glucocorticoid metabolism, and enhanced adipocyte mineralocorticoid receptor activity. It is unknown whether these changes in adrenal endocrine function are in part responsible for the pathogenesis of obesity and related comorbidities or represent an adaptive response. In turn, adipose tissue hormones or “adipokines” have direct effects on the adrenal glands and interact with adrenal hormones at several levels. Here we review the emerging evidence supporting the existence of “cross talk” between the adrenal gland and adipose tissue, focusing on the relevance and roles of their respective hormones in health and disease states including obesity, metabolic syndrome, and primary disorders of the adrenals.

## 1. Introduction

The significance of the adrenal gland as an endocrine organ and the roles of adrenal hormones in physiology and disease have been recognized well over a century [1, 2]. In fact, the history of adrenal endocrinology is almost as old as the study of endocrinology itself. Conversely, adipose tissue was long regarded as a hormonally inactive storage site for triglycerides. Only in recent years, paralleling the emergence of obesity and associated disorders such as diabetes, cardiovascular disease, and cancer as leading causes of morbidity and mortality has adipose tissue achieved recognition as an active endocrine organ playing key roles in maintaining homeostasis and involved in the pathogenesis of a variety of diseases [3–5].

The adrenal gland is composed of an outer cortex producing steroid hormones including mineralocorticoids, glucocorticoids, and sex steroids and an inner medulla of neuroectodermal origin producing catecholamine hormones. Each of these hormones acts on adipose tissue, which is readily apparent from the well-described clinical manifestations of primary adrenal endocrinopathies such as Cushing syndrome, Addison disease, and pheochromocytoma.

Adipose tissues are a heterogeneous group of tissues classified based on histology and function as white adipose tissue (WAT) and brown adipose tissue (BAT). Adipose tissues can be further categorized by anatomical location as visceral adipose tissue (VAT) and subcutaneous adipose tissue (SAT) [6]. While rodents maintain both BAT and WAT in distinct depots through adult life, BAT in humans is found mainly in the neck and thorax in neonates and is mostly replaced by WAT by adulthood [6].

The adipose tissues are a source of several hormones termed “adipokines” or “adipocytokines” including adipocyte-derived factors such as leptin, adiponectin, resistin, and proinflammatory cytokines such as tumor necrosis factor-**α** (TNF-**α**), C-reactive protein (CRP), serum amyloid A (SAA), and interleukin-6 (IL-6) which are secreted not only from adipocytes, but also from myeloid-derived cells such as macrophages located in the adipose tissue stroma [7, 8]. While adipokine roles in regulating systemic energy homeostasis, body weight, vascular biology, inflammation, and glucose and lipid metabolism have been well defined, more recently their regulation by adrenal hormones and effects on adrenal function has become a “hot topic” with increased representation in the scientific literature [9–11].

The growing body of knowledge describing two-way interactions between adrenal hormones and the “endocrine adipocyte” has resulted in the proposal of the existence of an “adipose-adrenal axis” [12]. In this review we critically examine the available published data investigating the interplay between the adrenals and adipose tissue with an emphasis on novel mechanistic insights into roles in pathophysiology of obesity and primary diseases of the adrenals.

## 2. Mineralocorticoid-Adipose Interactions

Aldosterone is the primary mineralocorticoid hormone produced by the adrenal glands in humans and exerts its effects via the mineralocorticoid receptor (MR). While the well-described “classical” effects of aldosterone on transepithelial Na transport are mediated by MR present in epithelial cells, MR has been shown to be present in a number of other cell types including adipocytes [13].

Activation of MR appears to have important roles in adipose tissue including differentiation of preadipocytes to mature adipocytes and promotion of a proinflammatory state via induction of cytokines including TNF-**α**, monocyte chemotactic protein-1 (MCP-1), and IL-6 in WAT, while decreasing the thermogenic activity and lowering uncoupling protein 1 (UCP1) transcription of BAT [14]. MR mRNA expression was shown to correlate with increasing body mass index (BMI) in humans and to be increased in obese db/db mice [15].

### 2.1. Mineralocorticoid Receptor Effects in Adipocytes Are Regulated Primarily by Glucocorticoids

**Figure 1:**
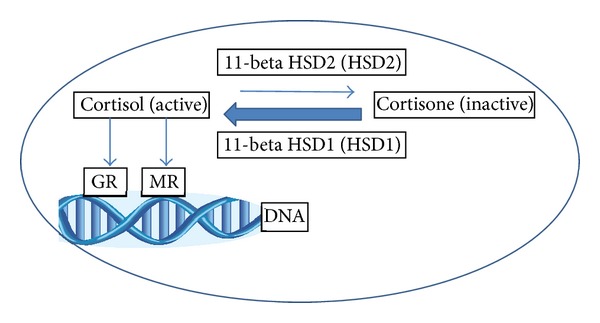
Adipocytes do not have significant HSD2 activity and yet maintain a relatively high level of HSD1 activity, allowing for increased intracellular active cortisol relative to inactive cortisone. Cortisol exerts its effects in adipocytes by binding to both glucocorticoid receptor (GR) and mineralocorticoid receptor (MR) with similarly high affinity. Obesity has been associated with increased adipose HSD1 activity, further increasing intra-adipocyte cortisol concentrations.

Since glucocorticoids (cortisol in humans and corticosterone in rodents) and mineralocorticoids (aldosterone) both bind to MR with relatively high affinity, the MR specificity of aldosterone in epithelial cells in humans is due to the intracellular inactivation of cortisol to cortisone (and corticosterone to 11-dehydrocorticosterone in rodents) by the enzyme 11-beta-hydroxysteroid dehydrogenase type 2 (11HSD2) (Figure 1). Adipocytes do not exhibit significant 11HSD2 activity, allowing glucocorticoids (GC), which circulate at 10-to-100-fold higher concentrations than mineralocorticoids (MC), to be the main ligand for adipocyte-MR [16]. Further enhancing the role of glucocorticoid hormones on adipose tissue MR activity is the increased 11-beta-hydroxysteroid dehydroxygenase type 1 (11HSD1) present in adipocytes, which results in increased conversion of cortisone to cortisol (Figure 1). While this balance of HSD enzyme activity explains the increased exposure of adipocyte-MR to endogenous glucocorticoids, the fact that glucocorticoid induced alteration in adipocyte function is an MR mediated phenomenon was demonstrated by studies in which adipocyte dysfunction induced by cortisol and corticosterone treatment in cultured murine adipocytes was reversed by the MR antagonist eplerenone but not by glucocorticoid receptor (GR) antagonism with mifepristone [15]. Therefore it is likely that many of the functions of glucocorticoids in regulation of adipocyte biology may be mediated via MR rather than GR.

### 2.2. Aldosterone in Obesity

Increased serum aldosterone levels have been observed with both obesity-associated hypertension and the metabolic syndrome. While several mechanisms may be at play, this finding is most likely due in large part to the upregulation of the renin-angiotensin-aldosterone system (RAAS) in obesity, a proposed mechanism linking hypertension with obesity that is reversed by weight loss [17].

A second, more novel mechanism of increased aldosterone production in obese individuals, exemplifying the concept of adrenal-adipose “cross talk,” is the identification of adipose-derived factors able to directly stimulate adrenal aldosterone production [12]. This finding raises the prospect of a “vicious cycle” seen in obese states between the adipocyte and adrenal zona glomerulosa in which adipose tissue derived factors could stimulate aldosterone production, leading to hypertension, inflammation, and endothelial dysfunction. The enhanced MR activation that results would in turn promote adipocyte differentiation and inflammation, promulgating the cycle. The exact nature of the adipocyte-derived factors is unknown, yet certain candidate fatty acids have been proposed, particularly oxidized derivatives of linoleic acid [18].

A third candidate mechanism contributing to our understanding of the proposed role of MR activation in adipose tissues was brought up by a recent report demonstrating both in vitro production and in vivo production of aldosterone by adipocytes, resulting in both autocrine and paracrine regulatory effects on adipocyte function [19].

Clinical implications of these insights into the role of adipocyte MR in obesity include whether MR blockade would be of benefit in treating obesity-associated morbidities such as hypertension, diabetes, and the metabolic syndrome. While studies of MR blockade in obese mice have shown benefits on adipose tissue dysfunction [15, 20], clinical trials in humans of MR blockers specifically investigating its effects in obesity do not exist. In clinical trials assessing effects on hypertension, RAAS blockade has been linked to decreased risk of new onset diabetes [21]. While the exact mechanisms that explain this observation are unknown, effects on adipocyte differentiation and inflammation have been proposed. Studies of MR blockade in cardiovascular disease have shown benefits, though not specifically on BMI or metabolic syndrome per se [22, 23].

### 2.3. Adipose Tissue Dysfunction in Primary Hyperaldosteronism

Primary hyperaldosteronism (PA), known also by its eponymous name Conn syndrome, is a state of continuous aldosterone excess due to autonomous production of aldosterone by an adrenal adenoma or bilateral adrenal zona glomerulosa hyperplasia and thus provides a relevant model for investigating the results of systemic aldosterone excess on adipose tissue.

While continuous infusion of aldosterone over 12 days resulted in weight gain in adrenalectomized rats [24], both cross-sectional studies and longitudinal studies of PA in humans, including those assessing changes following surgical cure of PA, report no consistent effect on body weight [14]. Though a relationship with increased insulin resistance and hyperglycemia has been described in older reports [25], several recent studies have not confirmed this finding [14]. While most studies of PA have not reported changes in circulating leptin and adiponectin, levels of the proinflammatory adipokine resistin were significantly increased in PA when compared to hypertensive controls and correlated with presence and severity of metabolic syndrome in PA subjects [26].

## 3. Glucocorticoid-Adipose Interactions

Glucocorticoids have effects on adipose tissue development, metabolism, and adipocyte secretory function. Studies investigating the effects of GC on adipogenesis and adipocyte lipid metabolism provide discordant results, while the anti-inflammatory nature of GR activation in adipocytes is a consistent finding [27, 28]. Glucocorticoids promote differentiation of preadipocytes to mature adipocytes acting synergistically with insulin [29]. In BAT, GC decrease UCP1 expression and increase lipid storage, in effect mediating a phenotypic conversion of BAT to WAT [30]. The effects of GC on adipose tissues particularly on lipid synthesis and lipolysis appear to depend on the physiologic context in which they occur as well as the specific depot, with predominantly lipogenic effect in VAT and lipolytic role in SAT [28]. Particularly important in determining the net effect of GC exposure on adipose lipid metabolism are nutritional and hormonal milieu. For instance while cortisol synergistically may stimulate adipocyte expansion during energy surplus and abundant insulin supply, such as what would occur in Cushing syndrome, during catabolic states cortisol production increases as part of the stress response and has a largely lipolytic role, mobilizing vital energy stores. This paradigm may partially explain the observation that hypercortisolism is associated with increased adiposity in Cushing syndrome and paradoxically with decreased adiposity in states of undernutrition such as anorexia and acute illness.

Alterations of circulating cortisol dynamics, mainly as hypothalamic-pituitary-adrenal (HPA) axis dysfunction, as well as local metabolism of glucocorticoids in adipose tissue, have been linked to obesity and the metabolic syndrome.

### 3.1. Adipocyte Cortisol Metabolism in Obesity

The dramatic changes in fat distribution characterized by central adiposity with wasting of subcutaneous fat depots, typical of Cushing syndrome, have led to the hypothesis that alterations in GC action may play a role in more common forms of visceral obesity. While circulating cortisol levels are not increased in obese individuals, increased net cortisol production occurs locally in the adipose tissue due to the above-described alterations in adipocyte HSD enzymes, and thus obesity has been described as “Cushing syndrome of the omentum” [31] (Figure 1).

An important confounder in interpreting studies of GC action on adipose tissue is the fact that GC effects in adipose tissue are mediated via the MR receptor as well as the GR receptor. Due to the lack of adipocyte activity of 11HSD2, an enzyme which inactivates endogenous GC in mineralocorticoid target tissues, much of the observed effect of naturally occurring GC in adipocytes is due to its effect on MR, rather than GR [32] (Figure 1).

Many studies have suggested increased 11HSD1 activity in obesity [33, 34]. Transgenic mice overexpressing 11HSD1 develop metabolic syndrome, while mice deficient in 11HSD1 are protected from metabolic syndrome and associated cardiovascular disease [35, 36]. A variety of studies in humans have found increased 11HSD1 in both VAT and SAT with obesity [37]. Several naturally occurring and synthetic HSD1 inhibitors have been investigated in regard to their effects on metabolic disorders [38]. One such compound, INCB13739, when given to subjects with T2DM improved HbA1c, insulin resistance, and lipid parameters. The effects were more pronounced in the obese group (BMI > 30) when compared with the overweight group (BMI > 25) [39]. These findings point to the importance of HSD enzymes in determining the effect of GC in adipose tissue and make HSD modification an attractive candidate for pharmacological intervention in the treatment of obesity.

While studies of the effects of GC on adipogenesis and adipocyte lipid metabolism have yielded inconsistent results, the effects of GC in suppressing inflammatory signaling from adipocytes are remarkably consistent. Experiments of selective GR stimulation with dexamethasone have generally resulted in decreased expression of proinflammatory cytokines by adipocytes, while GR knockdown with siRNA has the opposite effect [40]. Investigations of GC effects on adipocytokine production have yielded consistent findings of increased leptin expression [41] and inconsistent results regarding effects on adiponectin [42].

### 3.2. Obesity and the HPA Axis

Obesity and metabolic syndrome are associated with alterations in the HPA axis including changes in adrenal cortisol production, peripheral cortisol metabolism, and dynamic tests of the HPA axis. Whether these changes play a role in the development and pathophysiology of obesity and related comorbidities or represent an adaptation to the obese state is unknown. Basal levels of ACTH and cortisol appear to be undisturbed in obesity. While 24-hour urine-free cortisol generally has not been found to be increased in obese subjects, one study described significantly elevated night time (7 pm–7 am) urine-free cortisol in women with abdominal obesity [43]. The most convincing evidence of abnormal HPA axis function in obesity comes from dynamic studies of stimulation or suppression of the axis. Obese individuals exhibit greater ACTH and cortisol responses to AVP and CRH [44, 45] and increased cortisol response to both low dose and high dose ACTH stimulation tests [46, 47]. Though most studies have demonstrated normal cortisol suppression after 1 mg overnight dexamethasone suppression in obese subjects compared to lean controls, one study positively correlated higher postdexamethasone serum cortisol levels with increased waist-to-hip ratio in women, but not in men [48].

## 4. Adrenal Androgens and Adipose Tissue

The adrenal cortex produces androgen hormones in both sexes, including dehydroepiandrosterone (DHEA), dehydroepiandrosterone sulfate (DHEA-S), and androstenedione. In humans DHEA-S circulates at higher concentrations than any other steroid hormone and levels decline substantially with age, paralleling the changes in body composition characteristic of aging [49]. Of particular relevance is the fact that DHEA is present at nearly 10-time higher concentration in adipose tissue than in the systemic circulation [50]. A number of epidemiologic studies have shown correlations between circulating DHEA-S and obesity and insulin resistance and cardiovascular disease [51]. Both in vitro and in vivo studies have demonstrated antiadipogenic effects of DHEA on animal adipocytes [52]. DHEA effects favorable changes in adipocyte insulin sensitivity and adipokine profile [53, 54]. While several studies of DHEA supplementation in adults with various disease states have not shown consistent changes in body weight or body fat percentage [55], one study of DHEA administration in the elderly resulted in a significant decrease in abdominal fat after 6 months of treatment [56].

A potential confounder in interpreting these data is the fact that DHEA acts as a precursor hormone and is converted into both testosterone and estrogens, which have independent effects on adipose tissue and body composition [57]. Receptors for sex steroids are distributed differently among the genders and have important effects in regulating both lipoprotein lipase and leptin production [58, 59].

## 5. Adipose Tissue and the Adrenal Medulla

The adrenal medulla functions similarly to a sympathetic ganglion, secreting catecholamine hormones, primarily epinephrine, into the bloodstream under the control of the sympathetic nervous system (SNS). Epinephrine has potent lipolytic effects on adipose tissue [60] and impairment in catecholamine-dependent lipolysis has been implicated in the pathogenesis of obesity [61].

There is increasing data indicating cross talk between catecholamines and adipokines, with evidence supporting stimulation of adrenal medullary function by leptin, while resistin may suppress medullary catecholamine secretion [62]. Closing this adrenal-adipose feedback loop, catecholamines have been shown in vitro to modulate adipocyte endocrine function, promoting expression of proinflammatory cytokines by adipocytes and reducing leptin and resistin expression [62].

### 5.1. Adrenal Medullary Function in Obesity

Obesity is associated with overactivity of the sympathetic nervous system. This SNS “overdrive” has been implicated as a possible contributor to the pathogenesis of obesity-associated morbidities and end-organ damage attributed to the obese state. One theory explains the increased SNS output of obesity as a homeostatic counterregulatory mechanism aimed at redirecting excess energy supply into adrenergic thermogenesis, thus protecting from the harms of further fat storage [63]. Adipose tissue may play an important role in coordinating this response by secreting adipokines which stimulate SNS and adrenal medullary function [64]. Adipokines that may directly or indirectly modulate SNS activity include leptin, nonesterified-free fatty acids, angiotensinogen, TNF-**α**, IL-6, and adiponectin.

The relationship between leptin and catecholamine production may be of particular significance in the discussion of obesity as leptin has been implicated as a direct inducer of increased SNS outflow in obesity [65]. Conversely, acute activation of beta-adrenergic receptors has been shown to decrease leptin production by adipocytes in both humans and rodent models, though in a study of pheochromocytoma (PHEO) circulating leptin levels were not suppressed [66].

### 5.2. Catecholamines and BAT

Recent reports have highlighted the importance of catecholamines in regulating BAT in adults. Several observational studies of patients harboring PHEO describe that active BAT, as evidenced by increased uptake on 18-fluorodeoxyglucose-positron emission tomography (FDG-PET) by BAT, is diminished following surgical treatment of pheochromocytoma [67, 68]. Intriguing findings of a recent study were that adiponectin expression was increased from BAT in patients with PHEO while serum adiponectin levels decreased after surgical removal of PHEO and its surrounding BAT [69].

## 6. Nonfunctioning Adrenal Adenomas and Adipose Tissue

Adrenal masses are discovered in approximately 4% of CT or MRI studies performed for reasons other than suspicion of adrenal disorder [70]. Such incidentalomas are most often nonfunctioning adenomas (NFA) of the adrenal cortex, though 5–9% may result in mild hypercortisolism not resulting in clinically obvious Cushing syndrome, an entity defined as subclinical Cushing syndrome (SCS) [70]. NFA has been associated with obesity as well as insulin resistance and the metabolic syndrome. In reports comparing persons with NFA to obese controls matched for BMI, NFA subjects typically exhibit a more central distribution of fat associated with decreased insulin sensitivity [71]. In line with these findings, we previously reported increased epicardial fat thickness and left ventricular mass in NFA subjects [72]. Careful analysis of many of these studies reveals statistically significant higher indices of cortisol production, particularly late night serum cortisol and dexamethasone suppressed cortisol levels, but not to the degree meeting accepted criteria for SCS [71, 73]. Since cortisol production can be best understood as a continuous spectrum, one explanation for the abnormalities in adiposity and metabolic parameters observed in NFA patients could be subtle hypercortisolism. Others have proposed that NFA may be the result, rather than the cause, of insulin resistance [74].

## 7. Adipocytokine Effects on Adrenal Function

While the primary regulation of adrenal hormone production, classical glucocorticoids and androgens by ACTH, aldosterone by the RAAS, and catecholamines by the SNS, has been well described, it has more recently been proposed that adipokines may have a role in modulating adrenal function. While a comprehensive review of the effects of all adipokines on adrenal function is beyond the scope of this review, we will summarize the effects of two adipocytokines of particular relevance, leptin and adiponectin, as follows.

## 8. Leptin-Adrenal Interactions

Since its discovery as a WAT-derived circulating satiety factor in 1994 [75], the effects of leptin on adrenal function have been studied extensively. In vitro experiments have elucidated the direct effects of leptin on adrenal steroidogenesis. In bovine adrenal cell culture, leptin administration downregulated expression of several steroidogenic enzymes and blunted the cortisol response to ACTH [11, 76]. Similar results were found in studies of human and rat primary adrenal cell culture in which leptin inhibited ACTH-stimulated cortisol production but had no effect on basal cortisol production [77]. Leptin also appears to have effects at higher levels of the HPA axis. Leptin deficient ob/ob mice exhibit increased HPA activity [78]. In vivo experiments of chronic leptin administration in ob/ob mice demonstrate a blunting of the corticotropin-releasing hormone (CRH) response to stress and decreased basal corticosterone levels [78]. However, a study of short term administration of leptin in rhesus macaques yielded conflicting results demonstrating no effect of leptin on either basal or stimulated cortisol levels [79].

While the reciprocal relationship between leptin and sympathetic nervous system activity has been detailed above, leptin has also been shown to exert direct effects on the adrenal medulla. Leptin stimulates secretory activity of porcine chromaffin cells [80], while both resistin and leptin promoted secretion of catecholamines from rat chromaffin cells [62]. The same investigators demonstrated evidence of a reciprocal regulation of leptin by the adrenal medulla, providing experimental proof of catecholamine inhibition of secretion of leptin from cultured adipocytes, once more supporting the existence of an adipoadrenal axis.

## 9. Adiponectin-Adrenal Interactions

Adiponectin is the most abundant adipokine in the circulation with insulin-sensitizing, antiatherogenic and anti-inflammatory properties mediated mainly by the adenosine monophosphate-activated protein kinase (AMPK) and peroxisome proliferator-activated receptor gamma (PPAR*γ*) signaling pathways. Adiponectin receptors are present in both human and mouse adrenals [81, 82]. Paschke and colleagues reported that adiponectin receptors were present in all layers of the adrenal cortex and medulla in rats, while adiponectin mRNA expression was demonstrated in the rat adrenal zona glomerulosa only [10]. Short term exposure of the murine adrenocortical Y-1 cell line to adiponectin downregulated key enzymes of steroidogenesis, resulting in decreased corticosterone and aldosterone production [82]. In turn, both glucocorticoids and ACTH are known to decrease adiponectin production in WAT [83, 84]. In a primary culture of rat adrenocortical cells, treatment with adiponectin enhanced adrenocortical cell proliferation and increased corticosterone secretion in a dose-dependent manner while aldosterone secretion was unaltered [10].

## 10. Conclusions and Future Perspectives

The emergence of obesity as a worldwide epidemic has brought the study of adipocyte biology and secretory functions of adipose tissue to the forefront of biomedical and endocrine research. The interaction between adipokines and other endocrine organs is an area of recent intensive investigation. In this regard, the hormones of the adrenal glands are particularly relevant given their well-established effects on lipid metabolism, inflammation, and the “stress” response. Here we have discussed the physiologic and pathophysiologic actions of each of the adrenal hormones in the adipose tissues with focused discussions of the specific alterations observed in obesity and metabolic syndrome as well as mechanistic considerations of their roles in obesity and associated comorbidities. We have also examined the evidence linking adipocyte dysfunction to primary adrenal disorders and elucidated several examples of two-way interactions or cross talk between hormones of the adrenals and adipokines. Further research is needed to improve our collective understanding of the significance of the described changes in adipocyte function in primary adrenal diseases and particularly of the changes in adrenal function resulting from the adipokine milieu characteristic of obesity; a fundamental concept in need of further exploration is whether these are adaptive mechanisms representing a protective response to the obese state or, conversely, contributors or “drivers” in the morbidity associated with excess adiposity. Lastly, we have provided a theoretical basis for potential future pharmacological interventions aimed at adrenal hormone targets in the adipose tissue.
